# Bioactivity of miltefosine against aquatic stages of *Schistosoma mansoni, Schistosoma haematobium *and their snail hosts, supported by scanning electron microscopy

**DOI:** 10.1186/1756-3305-4-73

**Published:** 2011-05-11

**Authors:** Maha M Eissa, Samia El Bardicy, Menerva Tadros

**Affiliations:** 1 Department of Medical Parasitology, Faculty of Medicine, Alexandria University, Alexandria, Egypt; 2 Department of Medical Malacology, Theodor Bilharz Research Institute, Imbaba, Cairo, Egypt

## Abstract

**Background:**

Miltefosine, which is the first oral drug licensed for the treatment of leishmaniasis, was recently reported to be a promising lead compound for the synthesis of novel antischistosomal derivatives with potent activity *in vivo *against different developmental stages of *Schistosoma mansoni*. In this paper an *in vitro *study was carried out to investigate whether it has a biocidal activity against the aquatic stages of *Schistosoma mansoni *and its snail intermediate host, *Biomphalaria alexandrina *, thus being also a molluscicide. Additionally, to see whether miltefosine can have a broad spectrum antischistosomal activity, a similar *in vitro *study was carried out on the adult stage of *Schistosoma haematobium*, the second major human species, its larval stages and snail intermediate host, *Bulinus truncutes*. This was checked by scanning electron microscopy.

**Results:**

Miltefosine proved to have *in vitro *ovicidal, schistolarvicidal and lethal activity on adult worms of both *Schistosoma *species and has considerable molluscicidal activity on their snail hosts. Scanning electron microscopy revealed several morphological changes on the different stages of the parasite and on the soft body of the snail, which further strengthens the current evidence of miltefosine's activity. This is the first report of mollusicidal activity of miltefosine and its *in vitro *schistosomicidal activity against *S.haematobium*.

**Conclusions:**

This study highlights miltefosine not only as a potential promising lead compound for the synthesis of novel broad spectrum schistosomicidal derivatives, but also for molluscicidals.

## Background

Miltefosine (hexadecylphosphocholine) is one of several alkyllysophospholipid derivatives collectively known as alkylphosphocholines that were originally developed as anticancer agents [1]. The biocidal action of miltefosine against *Leishmania *species was demonstrated in the mid 1980s [2,3] and since then, trials for its clinical evaluation have led to the licensing of miltefosine for the oral treatment of leishmaniasis in India, Colombia, and Germany [4-6]. Miltefosine is also active against a variety of protozoa, and more and more data have become available on its activity against other Kinetoplastidae (*Trypanosoma cruzi *and *T. brucei*) [7,8], *Trichomonas vaginalis *[9], *Entamoeba histolytica *[10] and several free living amoebas [11-13]. Apart from its antiprotozoal effect, various bioactivities of miltefosine have been reported; it has a broad spectrum antifungal activity [14], bactericidal activity against *Streptococcus pneumoniae *and other pathogenic *Streptococci *[15], and it is under investigation as a potential therapy against HIV infection [16]. The mechanism underlying broad range bioactivities and the target (s) is still unrevealed. Most recently, miltefosine was reported to have anthelminthic properties. In a study done in 2011 [17], miltefosine was found to have schistosomicidal activity and showed comparative advantage over PZQ in being effective against in vivo differential developmental stages of *Schistosoma mansoni *in the mouse model.

Schistosomiasis is one of the most prevalent diseases in the world, with about 200 million human beings infected in 74 countries. It is estimated that 20 million of them have serious forms of the disease or related disabilities, and that 200,000 people die from the disease every year [18]. Chemotherapeutic measures have been the mainstay in the control of this disease [19]. Since 1970, praziquantel (PZQ) has become the drug of choice against the three major human species of schistosomes, *Schistosoma mansoni *(Sambon), *Schistosoma hematobium *(Bilharz), and *Schistosoma japonicum *(Katsurada) [20,21]. It is a relatively safe, orally administered drug that leads to reduction of the prevalence of schistosomiasis [22]. Consequently a targeted as well as mass drug administration program presently relies heavily on this drug for the control of schistosome-induced morbidity.

With only one drug of choice for treatment and the possibility of development of parasite resistance [23-27], the present situation is dangerous. Therefore, there is a real need for discovery of a new drug.

Though chemotherapy is one of the most effective methods for the control of schistosomiasis [28], there is a basic need for more selective and efficient molluscicides for the control of the snail vectors. The control of snails is an important means in the combat against this disease. The presently available synthetic molluscicides tend to be generally biocidal affecting many other animals and/or plants in the snail habitat [29]. Therefore, there is a need to search for other molluscicides with strong but specific activity and less harmful to the environment. As miltefosine, was reported to be a promising lead compound for the synthesis of novel anti-schistosomal derivatives with potent activity against in vivo different developmental stages of *S. mansoni *[17], this study was carried out to investigate whether a similar activity can also exist against its aquatic stages and its snail intermediate host, *Biomphalaria alexandrina*, thus being a molluscicide. In addition, to elucidate whether miltefosine can have a broad spectrum antischistosomal activity, a similar *in vitro *study was carried out on the same stages, the intermediate host, and the adult worm of *Schistosoma haematobium *the second major human species. Miltefosine efficacy was evaluated on basis of *in vitro *bioactivity testing supported by scanning electron microscopy.

## Materials and methods

### Drug

Miltefosine (Milteforan^®^) 2% veterinary oral solution was kindly supplied by Dr. Paolo Bianciardi, Scientific Advisor,Virbac, Italy.

### Bioactivity testing

#### *In vitro *schistosomicidal bioassay

The schistosomicidal *in vitro *bioassay used here followed the main procedure previously described by [30] and [31]. Thus, the schistosome material *Schistosoma mansoni *(Sambon) and *S. haematobium *(Bilharz) was obtained from the Schistosome Biological Supply Centre (SBSC), Theodor Bilharz Research Institute (TBRI), Cairo, Egypt. Mature worms were obtained from hamsters (*Mesocricetus auratus*), percutaneously infected with 350-400 *S. mansoni *cercariae per hamster 6-7 weeks earlier, and with *S. haematobium *cercariae 11-12 weeks earlier. The worms were obtained by perfusion using citrated saline, and the recovered worms were washed from blood in small sieves (20 μm mesh) by phosphate buffer. Worms were washed three times with the culture medium which is used for the assay under a sterilized laminar flow chamber. The culture medium used was RPMI 1640 + l-glutamine + 20% fetal calf serum + antibiotics (300μg streptomycin + 300 IU penicillin + 160 μg gentamycin per ml). The bioassay was carried out using 24 wells tissue culture plates. A stock solution 5 mg/ml of the compound was prepared in DMSO immediately before being used. Three pairs of worms, males and females equally were used for each test well in 2 ml medium and 2 replicats were set up for each species in each case. Exposure of worms to a standard concentration of 10 μg/ml of miltefosine (25 uM) was made for 5 days at 37°C ± 0.5°C in 5% CO2 incubator. A pure medium and a medium containing 0.5% of DMSO (vehicle) were used as negative controls, while praziquantel at 10 μg/ml was used as a reference drug. Worms were examined for their viability using a stereomicroscope, and those not showing motility for one minute were considered dead. The mortality rate of worms was calculated after 5 days exposure. The compound was then retested (secondary screen) using the same technique by successive descending dilutions of the solution. The mortality of worms was determined in each case and the LC_50 _and LC_90 _were calculated. The statistical program SPSS version 7.5 was used for the calculation.

### Larvicidal (ovicidal, miracidicidal and cercaricidal) activity

Eggs, miracidia and cercariae of *S. mansoni *and *S. haematobium *were also obtained from SBSC. The eggs were extracted from the intestines of infected hamsters (*Mescoricetus aurautus*). Miracidia were obtained from cleaned eggs by hatching them in small amounts of dechlorinated tap water. The cercariae were procured from experimentally infected *B. alexandrina *and *B.truncatus *snails at 25°C ± 2°C. The eggs, miracidia and cercariae of both *Schistosoma spp*. were exposed to the LC_50 _of miltefosine determined below on snails for 30 min., 25 min and 20 min respectively. Sinking down of the miracidia and cercariae with detachment of the tail in the later case give indication of death of these organisms.

### Molluscicidal tests

The snail material used were *Biomphalaria alexandrina *(Ehrenberg) and *Bulinus truncatus *(Audouin), the vectors of *S. mansoni *and *S. haematobium *in Egypt respectively. They were also obtained from the colonies maintained in SBSC. Adult *B. alexandrina *and *B. truncatus *snails 4-6 mm in diameter and 2-3 mm shell height respectively were used for testing the molluscicidal activity of miltefosine. The snails were fed on boiled lettuce leaves, blue green algae and fish food.

The efficacy of miltefosine against the adult snails was primarily determined using the standard method of World Health Organization recommendations [29]. Thus one liter of solution with a concentration 20 ppm was prepared and 10 snails were added. The snails were maintained in the solution for 24 h at 25°C + 2°C. After exposure, the snails were thoroughly washed and transferred to fresh water for another 24 h for recovery. Two replicas were carried out and two groups of snails were run in fresh dechlorinated water under the same experimental conditions as the negative control. The currently conventional molluscicide (Niclosamide) was used similarly as positive control. At the end of recovery period, the snails were examined for viability, and the dead snails were counted and recorded to calculate the mortality rate. Miltefosine was then retested by the same method using descending concentrations for LC_50 _and LC_90 _determination. The statistical program SPSS package version 7.5 was used for calculation.

### Scanning electron microscopy study

Eggs, miracidia, cercariae as well as adult worms of *Schistosoma mansoni*, and the soft body of its snail host, *B. alexandrina*, exposed to miltefosine, and non exposed samples that served as controls were fixed in a 10% glutaraldehyde and processed for examination by SEM [32].

### Ethical approval

All animal studies presented here have been approved by the local government based on national regulations for animal experimentation.

## Results & discussion

### *In vitro *schistosomicidal activity

Miltefosine showed 100% mortality of worms at 10 μg/ml after 5 days exposure. It was slightly more effective on adult worms of *S. haematobium *than on *S. mansoni *with LC_50 _of 5.1 μg/ml and 5.8 μg/ml for the two spp. respectively. However this effect is still much less than that of the reference drug (PZQ) which gave LC_50 _= 0.2 μg/ml after 5 days under the same condition (Table 1). This difference in susceptibility of the two species to miltefosine may be explained by one of the several hypotheses raised by Eissa *et al*., 2011 [17] for the possible mechanisms of action of miltefosine in schistosomiasis. They postulated that miltefosine may act through acetylcholine esterase inhibition due to the presence of phosphocholine moiety in its structure, a mechanism of action which is well known for the potent schistosomicidal drug, metrifonate, which was widely used against *S.haematobium *in the 1990s, and then withdrawn from the market because of medical, operational and economic criteria [33,34]. It is well known that *S. mansoni *and *S. haematobium *differ in their sensitivity to this therapeutic anticholinesterase, metrifonte, that exhibits activity against *S. haematobium *singly [35,36]. As Miltefosine was postulated to have anticholine esterase activity, we hypothesize that this may act synergistically with other postulated mechanisms raised for miltefosine by Eissa *et al*., 2011 [17], thus explaining the slight difference of susceptibility of the two species to miltefosine. However, further research is needed to provide a better understanding of the contribution of this factor in determining the different susceptibility of the two spp. to miltefosine.

**Table 1:** *In vitro *schistosomicidal activity of miltefosine on *Schistosoma mansoni *and *Schistosoma haematobium *adult worms (after 5 days exposure).

***Schistosoma *spp**.	Miltefosine		Praziquantel	
	
	LC_50_ μg/ml	LC_90_ μg/ml	LC_50_ μg/ml	LC_90_ μg/ml
*S.mansoni*	5.8	8.2	0.2	0.3

*S.haematobium*	5.1	7.1	0.2	0.3

**Figure 1:**
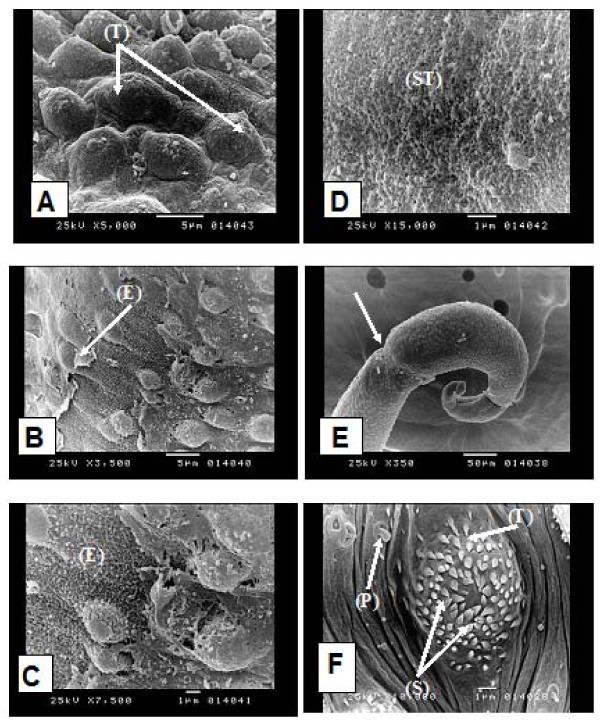
**Scanning electron microscopy (SEM) of adult *Schistosoma mansoni***: in vitro exposed adult to miltefosine showing: **(A) **distorted dorsal tegumental tubercles on the dorsal surface (T) (× 5000), **(B) **erosion of the tegumental surface (E) (× 3500), **(C) **higher magnification of (B) (× 7500), **(D) **appearance of subtegumental tissue (ST) (× 15000), **(E) **constriction at the posterior end of the worm (→) (× 350). Unexposed adult showing: **(F) **dorsal tegumental tubercle (T) of a normal male worm showing its spines (S) and papillae (P) (× 10000).

In the present study, SEM of adult male *S.mansoni *worm exposed *in vitro *to miltefosine at a concentration of 10 ug/ml (25 uM) showed distortion of the tubercles on the dorsal tegumental surface (Figure 1A). There was erosion of the tegumental surface (Figure 1B, C) with appearance of subtegumental tissue (Figure 1D). There was also constriction at the posterior end of the worm (Figure 1E). The normal dorsal tegumental tubercles from the negative controls (pure medium controls) are shown in (Figure 1F). SEM of worms from DMSO controls appeared similar to those from pure medium controls. SEM of the tegument of adult *S. mansoni *worms has been described since the seventies [37,38]. The present results show that miltefosine caused disintegration of the tubercles on the dorsal tegumental surface with its sloughing and erosion leading to exposure of the subtegumental tissue. These findings are similar but more pronounced than the changes observed by Eissa *et al*., 2011 [17] in their *in vivo *study. These results are also in accordance with tegumental alteration induced by mefloquine on adult *S. mansoni *in both *in vitro *and *in vivo *studies [39].

### Ovicidal, miracidicidal and cercaricidal activities

**Figure 2:**
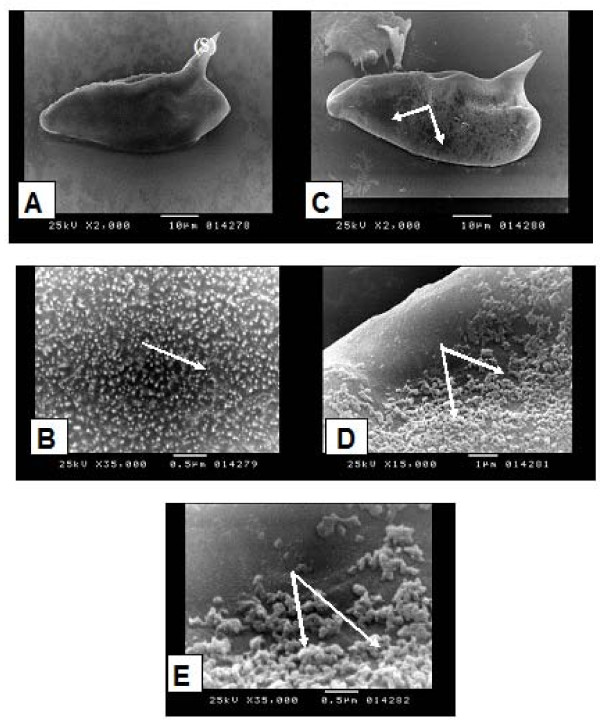
**Scanning electron microscopy (SEM) of *Schistosoma mansoni *eggs**: unexposed eggs showing: **(A)** oval egg with lateral spine (S) (× 2000), **(B)** the surface of the egg showed microspicules like chitinous projections (→) (× 35000). Exposed eggs to miltefosine showing: **(C)** patchy loss of the microspicules like chitinous projections (→) (× 2000), **(D)** higher magnification of (C) (× 15000), **(E)** swollen and oedematous chitinous projections (→) (× 35000).

Miltefosine proved to have clear schistolarvicidal effect on *S. mansoni *and *S. haematobium*. Thus eggs did not hatch at a concentration equal to LC_50 _of the drug on the corresponding snail vector (Table 2). However, the drug was more effective on *S. haematobium *than *on S. mansoni*. The compound leads to reduction in the movement of the miracidia and the sinking down of the cercariae to the bottom of the container with detachment of the tails. This was followed by the death of both organisms. Thus, this study showed also that miltefosine not only possesses the potential of being broad spectrum antischistosomal compound but also has schistolarvicidal activity against aquatic stages of both *S. mansoni *and *S. haematobium*. The biocidal activity of miltefosine was demonstrated against *S. mansoni *and *S. haematobium *eggs, leading to prevention of hatching when eggs were exposed to the mollusicicdal LC_50, _half and quarter this compound concentration. SEM study of *S. mansoni *egg showed that normal egg has microspicules like chitinous minute projections densely distributed all over the surface (Figure 2A& B). On the other hand, eggs exposed to miltefosine at a concentration of 3.75 ppm showed patchy loss of these projections (Figure 2C, D). The remaining ones became oedematous and swollen (Figure 2E). Previous literature showed that the egg shell of *S. mansoni *consists of dense material with multiple microspicules on its outer surface and pores through its thickness [40]. Within the shell is the live miracidium, which releases enzymes and antigens [41]. The present SEM of *S. mansoni *eggs after exposure to miltefosine showed destruction and patchy loss of the microspicules of the egg shell, which may indicates that miltefosine can diffuse easily and find its way inside the eggs. This, in addition to the porous shell of the egg that allows the drug to reach the metabolically active miracidium, where it may interfere with its antigenic substances and enzymes leading to the loss of their capability of hatching. Hatching of *Schistosoma *eggs is the culmulation of multiple biological processes and severe damage to several of these processes may be needed to be manifest as a decrease in the release of miracidia [40]. Further documenting this hypothesis is the observation of small granuloma size in the liver of experimentally infected mice with *S. mansoni *after treatment with miltefosine [17], that was explained by the authors due to the effect on eggs *in situ *by miltefosine which is known to accumulate in the liver after oral administration, thus diminishing the capacity of the eggs to induce delayed type hypersensitivity granulomas in the liver. Impairment of the antigenic products is clearly important to the host as morbid complications of schistosomiasis are largely related to the granulomatous and fibrotic responses to egg deposition in the tissues [42].

**Table 2:** Ovicidal and schistolarvicidal activity of miltefosine on *Schistosoma mansoni *and *Schistosoma haematobium*

***Schistosoma *spp**.	Concentration of Miltefosine Solution (μg/m l) Molluscidal LC_50_	Ovicicdal Effect	Miracidicidal Effect	Cercaricidal Effect
		
		100% mortality after (minutes)	100% mortality after (minutes)	100% mortality after (minutes)
*S. mansoni*	7.5	5	10	20
	
	3.75 (1/2)	10	15	30
	
	1.85 (1/4)	20	20	40
	
	0.925 (1/8)	30	30	50

*S. haematobium*	2.6	10	20	30
	
	1.3 (1/2)	20	30	45
	
	0.65 (1/4)	0	40	55
	
	0.33 (1/8)	0	60	60

**Figure 3:**
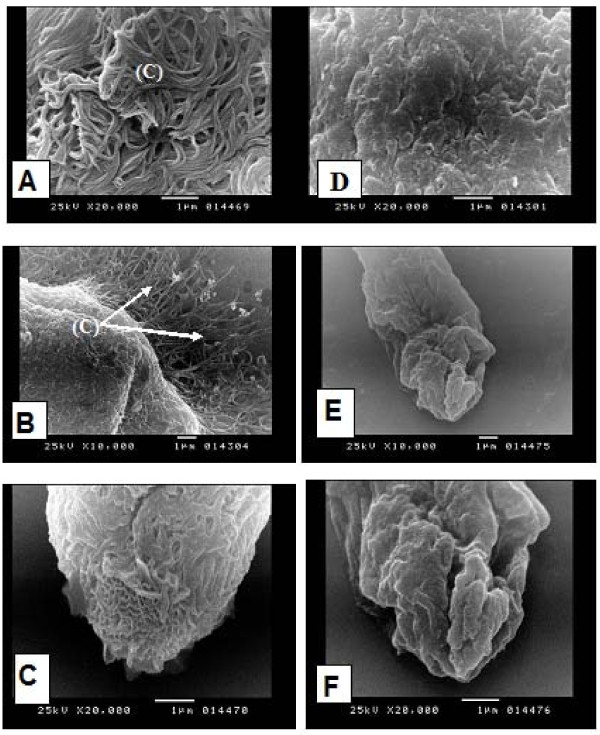
**Scanning electron microscopy (SEM) of *S. mansoni m*iracidia**, unexposed miracidia showing: **(A)** cilia (C) covering the surface of the miracidium (→) (× 20000), **(B)** cilia (C) covering the miracidium (→) and extending from its lateral side (× 10000), **(C)** the apical papillae of the miracidium with its characteristic honey comb pattern of its terebratoria (→) (× 20000). Miracidium exposed to miltefosine showing: **(D)** loss of cilia from the surface of miracidium (× 20000), **(E)** protruded apical papillae with swollen oedematous corregated areas (× 10000), **(F)** higher magnification of (E) (× 20000).

The present study showed also that miltefosine has biocidal activity against miracidia and cercariae of both *Schistosoma *species. Miltefosine leads to the reduction in movement of miracidia and sinking of the cercariae to the bottom of the container, this was followed by the death of both organisms. SEM studies showed that the normal unexposed miracidium of *S. mansoni *is covered with cilia (Figure 3A, B). The apical papillae showed the characteristic honey comb pattern of its terebratorium (Figure 3C). where as, the miracidium exposed to miltefosine at a concentration of 3.75 ppm showed distinct loss of cilia from its surface (Figure 3D) and the protruded apical papillae showed swollen oedematous corrugated areas (Figure 3E, F).

An SEM study of miracidia has been previously carried out by [43] and [44]. SEM of *S.mansoni *miracidium exposed to miltefosine showed loss of cilia from its surface, and the protruded apical papilla showed swollen oedematous corrugated areas. As a result the miracidium loses its ability to swim in water searching for its snail host. It may also lose its capability to penetrate the snails, because of the changes in the apical papilla [43].

**Figure 4:**
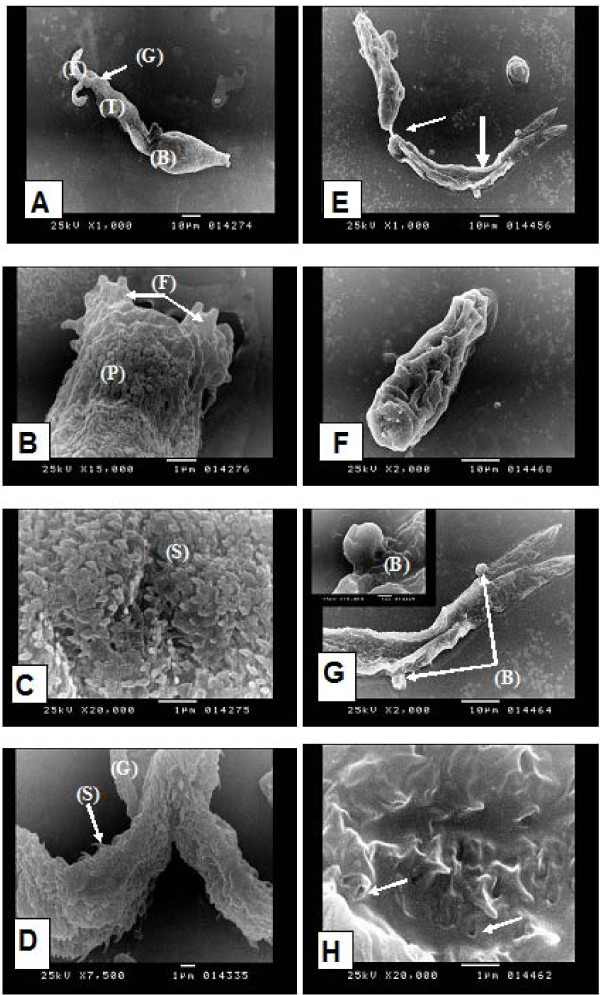
**Scanning electron microscopy (SEM) of *Schistosoma mansoni *cercariae**: normal cercaria showing: **(A)** cercarial body (B) and tail (T) with its two furculae (F), covered with glycocalyx (G) (× 1000), **(B)** tegumental folds (F) at the most anterior part of the head and evident tubular profiles (P) (× 15000), **(C)** numerous spines (S) covering the body region which are directed posteriorly and are covered with a glycocalyx that obscured these spines (× 20000), **(D)** the cercarial tail with its two furculae covered with glycocalyx (G) identical to that seen on the body but the tips of the spines (S) were often visible, larger and sharper than those of the body (× 7500). Cercaria exposed to miltefosine showing: **(E)** partial detachment of the body from the tail (→) with marked loss of the glycocalyx, thining of the tegument (→) and surface blebbing (× 1000), **(F)** separated cercarial body (× 2000), **(G)** surface blebbing of the tegument (B) (× 2000, inset × 15000). **(H)** focal loss of tegumental spines (→) (× 20000).

In the present study, SEM of unexposed cercariae showed that the cercarial glycocalyx envelops the whole organism (Figure 4A). The most anterior part of the head is provided with spiny tegumental folds (Figure 4B). At the body region the surface was irregular showing invagination and infolding of the tegument forming frequent tubular profiles (Figure 4B). The body is covered with numerous spines which are posteriorly directed and are covered with a glycocalyx that obscured these spines (Figure 4C). The cercarial tail and its furculae are covered by the glycocalyx similar to that seen on the body but the tips of the spines were often visible, larger and sharper than those of the body (Figure 4D). Cercariae exposed to miltefosine at a concentration of 3.75 ppm showed partial detachment of the body from the tail (Figure 4E), whereas in other cercariae, the body appeared completely separated from the tail (Figure 4F). There was marked loss of the glycocalyx, and thining of the tegument leading to external protrusion or surface blebbing (Figure 4E, G, inset), with focal loss of spines from the tegument (Figure 4H). SEM of *S. mansoni *cercariae has been described in a number of studies [45,46]. In the present study, *S. mansoni *cercariae exposed to miltefosine at concentration 3.75 ppm showed different morphological changes. Gross changes were observed, including loss of tails in about 80% of cercariae. These observations were consistent with the report of [47] and [48] which showed that hinokitiol (β- thujaplicin), a compound for potential skin application against cercarial penetration leads to loss of cercarial tail in about 50% of cercariae thus affecting cercarial movement and swimming activity. SEM also revealed loss of glycocalyx resulting in the thinning of the tegument causing external protrusion or surface blebbing with focal loss of spines. These changes are similar to the changes observed on the cercariae of *S.mansoni *exposed to hinokitiol by [48]. Those authors suggested that the structural changes may account for the inability of hinokitiol-treated cercariae to infect the host. Moreover, the surface blebbing is considered as an indicator for stress and has been observed in previous SEM studies evaluating anti-schistosomal drugs [49,39].

### Molluscicidal activity

**Figure 5:**
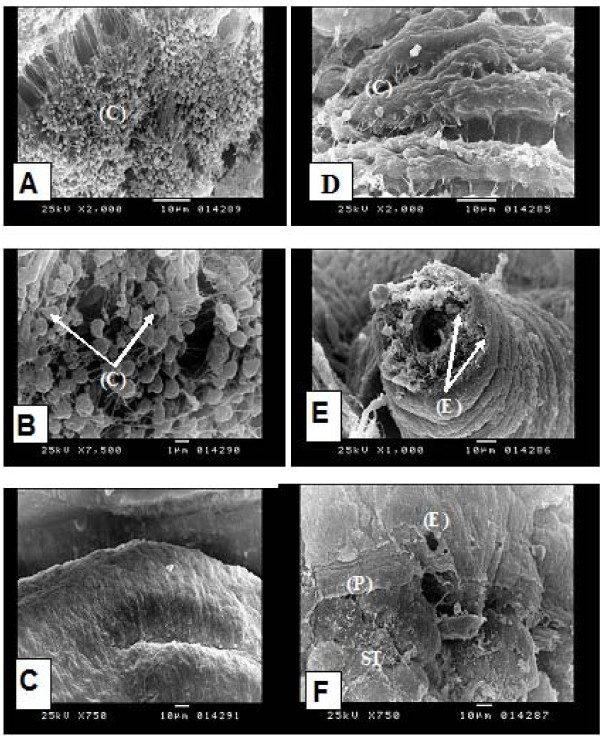
**Scanning electron microscopy (SEM) of *Biomphalaria alexandrina *snail (soft part)**: normal snail showing: **(A)** densly attached numerous cilia (C) at the ventral surface of the foot region (× 2000), **(B)** higher magnification of (A) showing cilia (C) (× 7500), **(C)** smooth tegumental surface of the mantle/visceral mass (× 750). Snail exposed to miltefosine showing: **(D) **extensive damage of the cilia at the foot region (C) (× 2000), **(E)** extensive damage of the tentacles with erosion (E) and exfoliation especially at its apical part with appearance of the subtegumental tissue (× 1000), **(F)** the mantle showed erosion (E), peeling (P) and exfoliation of its tegumental surface that results in exposure of the subtegumental tissue (ST) (× 750).

The results of this study showed that miltefosine has a considerable molluscicidal effect on both *B. alexandrina *and *B. truncatus *snails. *B. truncatus *is considrably more susceptible than *B. alexandrina*, LC_50 _for *Bulinus *was 2.6 ppm vs. 7.5 ppm for *Biomphalaria *after 24 h at 26°C. This molluscicidal effect is still much lower than that of niclosamide which is the conventional synthetic molluscicide commonly used at present, LC_50 _of niclosamide is 0.2 ppm under the same condition (Table 3). SEM of the unexposed soft body of *B. alexandrina *snail showed that the ventral surface of the foot is covered with cilia (Figure 5A, B) whereas, the tegumental surface of the mantle covering the visceral mass is almost smooth (Figure 5C). *B. alexandrina *snails exposed to miltefosine at concentration of 5 ppm showed extensive damage of the cilia at the foot (Figure 5D). There was also extensive damage of the tentacles with erosion and exfoliation especially at its apical part with appearance of the subtegumental tissue (Figure 5E). The mantle showed erosion, peeling and exfoliation of its tegumental surface that resulted in the exposure of the subtegumental tissue (Figure 5F). These changes are reminiscent of the morphological changes observed in scanning electron microscopy of tumor cells treated with miltefosine [50] and in adult *Schistosoma mansoni *after experimental in vivo treatment with miltefosine [17].

**Table 3:** Molluscicidal activity of miltefosine on *Biomphalaria alexandrina *and *Bulinus truncatus *snails after 24 hours at 25°C + 2°C.

Snail species	Miltefosine		Niclosamide	
	LC_50_ (ppm)	LC_90_ (ppm)	LC_50_ (ppm)	LC_90_ (ppm)
*B. alexandrina*	7.5	9.9	0.2	0.6
*B. truncates*	2.6	4.2	0.2	0. 6

A review of the literature showed that a number of studies have been carried out on the effect of several compounds and plant extracts on miracidia and cercariae as well as medically important snails with various degrees of success [51-54].

Although, the results of this study demonstrated the molluscicidal activity of miltefosine, its broad biocide activity may makes it unsuitable for snail control. Therefore, this study draws attention to miltefosine as a promising lead compound for the synthesis of more potent and selective molluscicidal derivatives.

## Conclusions

This study showed that miltefosine has a schistolarvicidal activity on the different aquatic stages of *S.mansoni *and *S.haematobium*, and a lethal *in vitro *effect on adult worm of both species. In addition, considerable molluscicidal activity was also demonstrated against their snail hosts. These biocidal activities were supported by SEM studies which further strengthen current evidence of miltefosine's activity. To the best of our knowledge, this is the first report of mollusicidal activity of miltefosine and its *in vitro *schistosomicidal activity against *S.haematobium*. Thus, this study highlights miltefosine not only as a potential promising lead compound for the synthesis of novel broad spectrum schistosomicidal derivatives but also for molluscicidals.

## Competing interests

The authors declare that they have no competing interests.

## Authors' contributions

MME: conceived and designed the research, performed the experiment and SEM study, literature search, wrote and revised the manuscript. SEB: designed the research, performed the experiment and SEM study, literature search, wrote and revised the manuscript. MT: performed the experiment, wrote and revised the manuscript.

All authors approved the final version of the manuscript.
